# Clinical Evaluation of Omadacycline for Pulmonary Mycobacterium *abscessus* Complex Infections: A Subgroup Analysis With a Focus on Macrolide Resistance

**DOI:** 10.1016/j.chpulm.2026.100238

**Published:** 2026-02-13

**Authors:** Mohammed Al Musawa, Amer El Ghali, Raaga Bean, Mehriban Mammadova, Carly Wadle, Anahit Muscarella, Christo Cimino, John Zeuli, Catessa Howard, Saira Butt, Carlos Mejia-Chew, Emily A. Kaip, Maria G. Tupayachi-Ortiz, Christina T. Fiske, Chloe Judd, Kaylee E. Caniff, Michael J. Rybak

**Affiliations:** a Anti-Infective Research Laboratory, Department of Pharmacy Practice, Eugene Applebaum College of Pharmacy and Health Sciences, Wayne State University, Detroit, MI; b University of Texas Health Science Center, University of Texas, Tyler, TX; c Division of Infectious Diseases, Department of Medicine, University of Texas Health Science Center at Houston, Houston, TX; d Vanderbilt University Medical Center, Nashville, TN; e Department of Pharmacy, Mayo Clinic, Rochester, MN; f West Virginia University Medicine Department of Pharmacy, Morgantown, WV; g Indiana University School of Medicine, Indianapolis, IN; h Division of Infectious Diseases, Department of Medicine, Washington University School of Medicine, St Louis, MO; i Department of Pharmaceutical Services, University of California, San Francisco Medical Center, San Francisco, CA; j Department of Medicine, Division of Pulmonary and Critical Care Medicine, Miller School of Medicine, University of Miami, Miami, FL; k Division of Infectious Diseases, School of Medicine, Wayne State University, Detroit, MI

**Keywords:** antibiotic therapy, macrolide resistance, *Mycobacterium abscessus* complex, nontuberculous mycobacteria, omadacycline, pulmonary infections

## Abstract

**Background:**

Pulmonary infections caused by *Mycobacterium abscessus* complex (MABC) are notoriously difficult to treat due to high levels of mutational and inducible antibiotic resistance. Omadacycline (OMC), an aminomethylcycline antibiotic with both oral and IV formulations, has shown in vitro activity against MABC and may offer a more tolerable alternative to conventional therapies. However, clinical data specific to pulmonary MABC with macrolide resistance remain limited. The aim of this study was to evaluate the clinical and microbiological outcomes of OMC-based therapy in patients with pulmonary MABC infections.

**Research Question:**

What are the clinical and microbiological outcomes of OMC-based therapy in patients with pulmonary MABC infections, including those with macrolide resistant isolates?

**Study Design and Methods:**

This was a retrospective subgroup analysis of a previously published multicenter cohort study conducted across 16 US academic medical centers. Adult patients with pulmonary MABC infections who received OMC for ≥ 72 hours and had ≥ 3 months of follow-up were included. The primary outcome was clinical success at 3, 6, and 12 months, defined as a composite of clinical improvement, survival, continued OMC use, and absence of microbiologic recurrence. Secondary outcomes included sputum culture improvement, outcomes by MABC subspecies and macrolide susceptibility, and adverse drug reactions.

**Results:**

Thirty-five patients were included (68.6% macrolide resistance). The median treatment duration was 8 months (interquartile range, 3.9-15.7). Clinical success was achieved in 71.4%, 89.7%, and 90.9% at 3, 6, and 12 months, respectively. Culture improvement occurred in 73.9% of evaluable patients, including 100% of those with macrolide-resistant strains. OMC was discontinued in 8 patients (22.9%) due to adverse drug reactions, most commonly gastrointestinal intolerance (26%) and transaminitis (11%).

**Interpretation:**

Our results show that OMC demonstrated high clinical success and culture improvement rates in pulmonary MABC, including macrolide-resistant cases. These findings support its potential role as a tolerable and effective oral agent within multidrug regimens. Prospective studies are needed to confirm these results and define optimal use strategies.

**Take-Home Points**

**Study Question:** What are the clinical and microbiological outcomes of omadacycline-based therapy among patients with pulmonary *Mycobacterium abscessus* complex (MABC) infections, particularly those caused by macrolide-resistant MABC?

**Results:** Clinical success was observed in 71.4%, 89.7%, and 90.9% of patients at 3, 6, and 12 months, respectively, and 73.9% of evaluable patients achieved sputum culture improvement; among patients with macrolide-resistant MABC, 86.7% achieved clinical success at 12 months, and all evaluable patients achieved sputum culture improvement.

**Interpretation:** These findings support omadacycline as a potential oral treatment option as part of multidrug therapy for pulmonary MABC infections, including infections caused by macrolide-resistant MABC, although prospective studies are needed to confirm these results and better define its role in treatment.

*Mycobacterium abscessus* complex (MABC) is a rapidly growing nontuberculous mycobacteria (NTM) that is increasingly recognized as a cause of serious and difficult-to-treat infections.^1^ These organisms are found in soil, natural and engineered water sources, and in medical devices.^2^ MABC is reported to be the second most common NTM responsible for pulmonary disease, especially in patients with underlying lung conditions such as cystic fibrosis, and bronchiectasis, and patients with gastroesophageal reflux disease.^2^^,^^3^

MABC includes the following 3 subspecies: *M abscessus*, *Mycobacterium massiliense*, and *Mycobacterium bolletii*; identifying the specific subspecies is crucial because it guides selecting the appropriate treatment.^2^ This is particularly important as both subspecies *M abscessus* and *M bolletii* possess a functional [*erm*(41)] gene, which can cause inducible resistance to macrolides in clinical settings. Additionally, subspecies *M abscessus* and *M massiliense* can develop mutational macrolide resistance.^1^

Current guidelines for MABC suggest that the selection of treatment agents should be guided by in vitro susceptibility testing.^4^ However, only macrolides and amikacin have validated minimum inhibitory concentrations.^5^ The limited treatment options due to intrinsic resistance to many commonly used antibiotics, including macrolides and beta-lactams, highlight the urgent need for new antimicrobial agents.^1^^,^^4^, ^5^, ^6^

Omadacycline (OMC) is an aminomethylcycline antibiotic that offers broad-spectrum activity and is available in both oral and IV forms.^7^ The US Food and Drug Administration has approved it for use in adults with community-acquired bacterial pneumonia and acute bacterial skin and soft tissue infections.^8^ In vitro studies have shown that OMC is effective against NTM pathogens including MABC.^9^, ^10^, ^11^ Like other tetracyclines, OMC works by binding to the 30S subunit of bacterial ribosomes, disrupting protein synthesis. However, it differs from earlier tetracyclines because it has been structurally optimized to overcome common resistance mechanisms, such as ribosomal protection and drug efflux.^7^

One of the main challenges in treating multidrug-resistant MABC is the limited availability of effective oral treatments, especially for macrolide-resistant strains.^4^^,^^5^ Additionally, tetracycline-based therapies often have poor tolerability, which can lead to treatment discontinuation due to adverse effects such as gastrointestinal upset and elevated liver enzymes. However, preliminary data suggest that OMC may be better tolerated than alternatives like IV tigecycline, with fewer associated side effects.^12^ The aim of this study was to evaluate the clinical and microbiological outcomes of OMC in patients with pulmonary infections caused by MABC with a focus on macrolide-resistant cases.

## Study Design and Methods

### Study Design and Data Source

This was a retrospective subgroup analysis derived from a previously published multicenter cohort study that evaluated the long-term effectiveness and safety of OMC in patients with NTM infections. The original cohort included 75 adult patients who received OMC at 16 academic medical centers across the US between January 1, 2020, and March 30, 2023.^13^ Given the clinical challenges associated with treating MABC infections, driven by limited available treatment options and multiple underlying resistance mechanisms, we conducted a focused subgroup analysis to specifically evaluate clinical outcomes, microbiological response, and drug tolerability among patients with MABC infections treated with OMC stratified by the macrolide susceptibility (either the presence of mutational resistance by antimicrobial susceptibility test or presence of a functional *erm* gene by molecular testing) and MABC subspecies.

### Patient Selection

Patients were eligible for inclusion if they met all the following criteria: (1) ≥ 18 years of age, (2) pulmonary infection caused by MABC (including subspecies *M abscessus*, *M massiliense*, or *M bolletii*), (3) received OMC for ≥ 72 hours, and (4) had available clinical follow-up for at least 3 months after treatment initiation. Patients were excluded if they were pregnant, incarcerated, or lacked follow-up data. Subspecies identification and susceptibility testing were performed at local laboratories in accordance with standard clinical practice.

### Data Collection

Clinical and demographic data were extracted from the parent cohort database.^13^ Collected variables included age, sex, race, insurance status, comorbidities, radiographic findings, MABC subspecies, *erm*(41) gene sequevar, mutational macrolide resistance, OMC treatment duration, concomitant antibiotic use, and adverse events. Culture improvement was defined as at least 2 consecutive negative sputum cultures collected ≥ 1 month apart within 12 months of OMC initiation. The reasons for OMC use (eg, prior treatment failure, oral availability, drug resistance) were also recorded.

### Outcomes

The primary outcome was clinical success at 3, 6, and 12 months, defined as a composite of the absence of microbiologic recurrence, clinician-evaluated clinical improvement, OMC continuity (no switch for failure or adverse effect), and survival. The secondary outcomes were clinical success by subspecies and macrolide susceptibility, sputum culture improvement in patients with positive cultures at the initiation of OMC therapy (defined as achieving ≥ 2 consecutive negative sputum cultures during OMC treatment), and adverse drug reactions (ADRs).

### Statistical Analysis

Descriptive statistics were used to summarize patient characteristics and outcomes. Categorical variables were expressed as frequencies and percentages, whereas continuous variables were reported as medians and interquartile ranges (IQRs). All statistical analyses were performed using SPSS version 30.0 (IBM Corp).

### Ethical Considerations

This secondary analysis was performed on deidentified data and was deemed exempt from further institutional review board oversight, consistent with federal guidelines for retrospective observational research.

## Results

### Baseline Characteristics

A total of 35 patients with MABC infections were included in this subgroup analysis (Table 1). The median age was 61 years (IQR, 51-70), and 74.3% were female patients. Most patients were White (74.3%) and were insured through government programs (60.0%). The median BMI was 22.5 kg/m^2^ (IQR, 19.3-25.4), with a median Charlson Comorbidity Index score of 3 (IQR, 2-4). Common comorbidities included the following: asthma (25.7%), immunosuppression (17.1%), and COPD (17.1%); 22.9% had no documented comorbidities.

**Table 1:** Baseline Characteristics (N = 35)

Variable	Value
Characteristic	
Age, y	61 (51-70)
Female	26 (74.3)
BMI, kg/m^2^	22.5 (19.3-25.4)
White	26 (74.3)
Insurance	
Government	21 (60.0)
Private	16 (45.7)
Charlson Comorbidity Index	3 [2-4]
Comorbid conditions^a^	
None	8 (22.9)
Asthma	9 (25.7)
Immunosuppression	6 (17.1)
COPD	6 (17.1)
Cystic fibrosis	5 (14.3)
Solid tumor	2 (5.7)
Connective tissue disease	2 (5.7)
Peripheral vascular disease	2 (5.7)
Diabetes	2 (5.7)
Leukemia	2 (5.7)
Others^b^	4 (11.4)
MABC subspeciation	
*Mabscessus*	17 (48.6)
*M massiliense*	3 (8.6)
*M bolletii*	2 (5.7)
Not determined	13 (37.1)
Other NTM species with MABC	10 (28.6)
*Mycobacterium avium* complex	8 (80.0)
*Mycobacteroides chelonae*	1 (10.0)
*Mycobacterium fortuitum*	1 (10.0)
MABC symptom criteria^a^	
Chronic cough	28 (80.0)
Fatigue	22 (62.9)
Weight loss (> 5%)	11 (31.4)
Hemoptysis	9 (25.7)
Night sweats	5 (14.3)
No. of symptoms	
1-2	23 (65.7)
3-5	12 (34.3)
Radiologic findings^a^	
Nodular	15 (42.9)
Tree-in-bud	17 (48.6)
Bronchonodular	13 (37.1)
Cluster of micronodules	10 (28.6)
Fibrocavitary	7 (20.0)
OMC duration, mo	8 (3.9-15.7)
Dissemination (before OMC initiation)	3 (8.6)
OMC loading dose (450 mg po once daily, day 1-2)	9 (25.7)
Tigecycline MIC, μg/mL (n = 27)	0.25 (0.25-0.5)
Concomitant antibiotic(s) with OMC^a^^,^^c^	
Amikacin (IV)	15 (42.9)
Imipenem (IV)	15 (42.9)
Clofazimine	15 (42.9)
Amikacin (inhaled)	15 (42.9)
Azithromycin	13 (37.1)
Linezolid/tedizolid	12 (34.3)
Clarithromycin	2 (5.7)
Ethambutol	2 (5.7)
Cefoxitin	2 (5.7)
Moxifloxacin/levofloxacin	2 (5.7)
Rifabutin	1 (2.9)
Reasons for OMC utilization^a^	
Previous antibiotic failure	18 (58.1)
Oral availability of OMC	14 (45.2)
Resistance to other agents	13 (41.9)
Ease of administration	13 (41.9)
Antimicrobial resistance to previous antibiotics	11 (35.5)
Oral step-down therapy	6 (19.4)
Patient preference	3 (9.7)
Antibiotic allergies	2 (6.5)
Renal failure with previous antibiotics	1 (3.2)

Data are reported as No. (%), mean [SD], or median (interquartile range). MABC = *Mycobacterium abscessus* complex; NTM, nontuberculous mycobacterium; OMC = omadacycline; MIC, miniumun.

a The numbers are not mutually exclusive.

b Other comorbidities include the following: cerebrovascular disease (n = 1), moderate to severe chronic kidney disease (n = 1), heart failure (n = 1), and peptic ulcer disease (n = 1).

c Concomitant antibiotics refer to any antimicrobial agents administered in combination with omadacycline for at least 48 h at any point during therapy. Azithromycin, when used, was part of background multidrug treatment.

MABC subspeciation was available for 62.9% of cases, with subspecies *M abscessus* accounting for 48.6%, followed by *M massiliense* (8.6%) and *M bolletii* (5.7%). Coisolation with other NTM species occurred in 28.6% of patients, most commonly *Mycobacterium avium* complex (80% of coinfections). Most patients (80.0%) reported chronic cough, and 62.9% reported fatigue. Radiographic findings were consistent with nodular and tree-in-bud patterns in 42.9% and 48.6% of cases, respectively. Median OMC treatment duration was 8 months (IQR, 3.9-15.7), with 25.7% receiving a loading dose. The most common reasons for OMC utilization were previous antibiotic failure (58.1%), oral availability of OMC (45.2%), and antimicrobial resistance to previous antibiotics (35.5%).

### Macrolide Susceptibility

Among all MABC cases, phenotypic or genotypic evidence of macrolide resistance was present in 68.6% (24 of 35), absent in 14.3% (5 of 35), and with no information available in 17.1% (6 of 35); mutational macrolide resistance was present in 75% (12 of 16) of the isolates tested. A functional *erm*(41) gene sequevar was detected in 92.3% (12 of 13) of the isolates that underwent testing. Among subspecies *M abscessus* cases, 76.5% (13 of 17) demonstrated macrolide resistance or harbored a functional *erm*(41) gene.

### Clinical and Microbiological Outcomes

Overall clinical success after OMC initiation was achieved in 71.4% (25 of 35) at 3 months, increasing to 89.7% (26 of 29) at 6 months and 90.9% (20 of 22) at 12 months (Table 2). Sputum culture improvement was achieved in 73.9% (17 of 23) of those who had positive culture when OMC was started. Among subspecies *M abscessus*, 3-, 6-, and 12-month success rates were 64.7%, 92.3%, and 77.8%, respectively. Patients with subspecies *M massiliense* and *M bolletii* demonstrated high success rates across all time points.

**Table 2:** Clinical Success and Microbiological Outcomes^a^

Study Group	3 mo	6 mo	12 mo	Sputum Cx. Improvement^b^
All MABC	25/35	26/29	20/22	17/23
By subspecies				
***M abscessus***	11/17	12/13	7/9	10/12
***M massiliense***	2/3	2/3	2/2	2/2
***M bolletii***	2/2	1/1	1/1	0/1
Not determined	10/13	11/12	10/10	5/8
By macrolide resistance^c^				
Macrolide-susceptible	4/5	4/4	4/4	0/0
Macrolide-resistant^d^	17/24	15/20	13/15	14/14
Unknown	4/6	5/5	3/3	3/3

Data are presented as No. of patients/total No. of patients. Cx = culture; MABC = *Mycobacterium abscessus* complex; OMC = omadacycline.

a Clinical success was defined as a composite of the absence of microbiological recurrence, clinician evaluated clinical improvement, OMC continuity (no switch for failure or adverse effect), and survival.

b Sputum culture improvement in patients with positive cultures at the initiation of OMC therapy (defined as achieving ≥ 2 consecutive negative sputum cultures during OMC treatment).

c Resistance classification (mutational, inducible, or susceptible) was based solely on phenotypic or genotypic testing before therapy and was not modified by the use of azithromycin.

d Macrolide-resistant includes both mutational (as defined by drug susceptibility test) and inducible (*erm*[41]) resistance.

When stratified by macrolide susceptibility, clinical success among patients with macrolide-resistant MABC (ie, includes both mutational and inducible [*erm*(41)] resistance) was 70.8% (17 of 24) at 3 months, 75.0% (15 of 20) at 6 months, and 86.7% (13 of 15) at 12 months. All evaluable patients with macrolide-resistant strains achieved sputum culture improvement (14 of 14). Among those with unknown macrolide susceptibility, clinical success ranged from 66.7% to 100% across time points, with culture improvement observed in 3 of 3 evaluable patients.

### Adverse Drug Reactions

ADRs were observed in a subset of patients receiving OMC. The most reported ADR was gastrointestinal intolerance, defined as nausea and vomiting, occurring in 26% of patients. Transaminitis (aspartate aminotransferase or alanine aminotransferase > 3 times the upper limit of normal) was observed in 11%, and headache was found in 6%. Less frequent ADRs included electrolyte disturbances, neutropenia, rash or dermatologic reaction, eosinophilia, and black tongue, each reported in 3% of patients (1 of 35). OMC was discontinued due to ADRs in 8 patients—4 due to transaminitis and 4 due to gastrointestinal intolerance. Overall, 66% of patients reported no adverse effects during treatment.

## Discussion

In this multicenter subgroup analysis of patients with pulmonary MABC infections, OMC demonstrated sustained clinical success and favorable microbiologic responses across a range of radiologic presentations, including patients with nodular, tree-in-bud, and fibrocavitary patterns. These real-world findings support the potential utility of OMC as part of multidrug therapy for pulmonary MABC, particularly when therapeutic options are constrained by resistance, toxicity, or oral administration needs.

This analysis expands on findings from the parent multicenter cohort by El Ghali et al,^13^ which assessed OMC across a broader NTM population that included skin and soft tissue, bone and joint, and other nonpulmonary sites. Our focused evaluation of 35 patients with confirmed pulmonary MABC demonstrated a 12-month clinical success rate of 90.9% and sputum culture improvement in 73.9% of evaluable patients. Of note, 100% of evaluable patients with macrolide-resistant MABC achieved culture improvement, highlighting OMC’s potential value in this high-risk subgroup.

Our findings differ from those reported in the larger retrospective study by Mingora et al,^14^ which primarily assessed the long-term safety and tolerability of OMC in 117 patients with MABC infections. Although that study documented an overall discontinuation rate of 51.3% and a culture improvement rate of only 18% among pulmonary cases, interpretation of these results is limited by several methodologic considerations. Notably, only a small proportion of patients classified as discontinuations (17.1%) had completed therapy as planned, whereas 19.7% discontinued because of ADRs or cost-related or nonclinical factors. Additionally, approximately 50% of patients remained on OMC at the time of data cutoff, resulting in incomplete information on treatment duration and outcomes. As the authors acknowledged, “a large proportion (∼50%) of the patients were still on therapy at the time of data analysis,”^14^ which likely contributed to the low culture improvement rate reported. Furthermore, culture improvement analysis was not restricted to evaluable patients, in contrast to our study where microbiologic outcomes were assessed exclusively among patients with positive cultures at OMC initiation.

In comparison, our analysis used clearly defined clinical and microbiologic end points, with final outcomes available for all patients included. This approach differs from the Mingora et al^14^ cohort, as previously described. We used a composite clinical success definition that encompassed clinician-assessed improvement, continued OMC use, patient survival, and absence of microbiologic recurrence, thereby providing a more comprehensive and clinically meaningful assessment of therapeutic response. These methodologic differences likely contributed to the higher clinical success and culture improvement rates observed in this cohort and underscore the necessity of standardized outcome definitions in future studies evaluating pulmonary MABC infections.

Regarding safety, gastrointestinal intolerance (26%) and transaminitis (11%) were the most common ADRs in this cohort, leading to treatment discontinuation in 8 patients. Other ADRs were infrequent and mild; notably, 66% of patients reported no adverse reactions. These safety outcomes compare favorably with prior reports and support the continued investigation of OMC in prolonged treatment regimens.^13^^,^^14^

This study is limited by its small sample size, retrospective design, and variability in data availability across centers. In particular, the timing of ADRs and the corresponding OMC dose at which they occurred were not consistently captured, which restricts our ability to characterize dose-related or time-dependent toxicity. Moreover, patterns of adjunctive antibiotic use also introduced additional complexity; patients received up to 12 different concomitant agents that were started and stopped at varying points during their treatment course. Because these adjunctive therapies differed widely in timing, duration, and combination, and because the number of patients receiving any specific regimen was small, we were unable to assess the independent impact of adjunctive therapy on clinical outcomes or ADRs.

However, this study contributes important real-world evidence supporting the efficacy, safety, and durability of OMC-based regimens in pulmonary MABC, including macrolide-resistant disease. Given the limited armamentarium for treating refractory MABC infections, particularly in outpatient settings, prospective trials should further investigate optimal OMC-based combinations, duration, and comparative effectiveness against alternative agents such as tigecycline or bedaquiline.

## Interpretation

OMC demonstrated promising clinical and microbiologic outcomes in patients with pulmonary MABC infections, including those with macrolide resistance. However, given the small sample size and retrospective design, these findings should be interpreted as preliminary and hypothesis-generating. OMC may represent a potentially useful and generally well-tolerated oral component of multidrug regimens for refractory pulmonary MABC, but its definitive role remains to be established. Larger, prospective studies are needed to confirm these observations, determine its optimal use within combination therapy, and assess long-term outcomes across diverse patient populations.

## Funding/Support

This investigation was supported by an investigator initiated grant from Paratek Pharmaceuticals.

## Financial/Nonfinancial Disclosures

The authors have reported to *CHEST Pulmonary* the following: M. J. R. has received grant support, has consulted, or has participated on speaker bureaus for Paratek, AbbVie, Innoviva, Melinta, Merck, and Shionogi. C. C. has received honorarium or served on an advisory board from Paratek and Shionogi. None declared (M. A. M., A. E. G., R. B., M. M., C. W., A. M., J. Z., C. H., S. B., C. M.-C., E. A. K., M. G. T.-D., C. T. F., C. J., K. E. C.).
